# Knowledge and beliefs on antimicrobial resistance among physicians and nurses in hospitals in Amhara Region, Ethiopia

**DOI:** 10.1186/2050-6511-15-26

**Published:** 2014-05-19

**Authors:** Bayeh Abera, Mulugeta Kibret, Wondemagegn Mulu

**Affiliations:** 1 Department of Microbiology, Parasitology and Immunology, College of Medicine and Health Sciences, Bahir Dar University, P.O. Box 79, Bahir Dar, Ethiopia; 2 Department of Biology, Science College, Bahir Dar University, P.O. Box 79, Bahir Dar, Ethiopia

**Keywords:** Antimicrobial resistance, Knowledge, Belief, Ethiopia

## Abstract

**Background:**

Antimicrobial resistance (AMR) is a major global public health problem both in hospital and community acquired infections. The present study assessed the knowledge and beliefs on AMR among physicians and nurses in 13 hospitals in Amhara region, Ethiopia, which is a low-income country.

**Methods:**

A cross-sectional study using a self-administered questionnaire was applied.

**Results:**

A total of 385 participants (175 physicians and 210 nurses) took part in the study. Sixty five percent of physicians and 98% of nurses replied that they need training on antimicrobial stewardship. Only 48% of physicians and 22.8% of nurses had exposures for local antibiogram data. Overall, 278 (72.2%) of participants were knowledgeable about AMR. Majority of participants agreed or strongly agreed AMR as worldwide and national problem but few considered AMR as problem in their own hospitals. The two most important factors mentioned for AMR development were patients’ poor adherence to prescribed antimicrobials (86%) and overuse of antibiotics (80.5%). The most leading local factors identified for AMR development were: self-antibiotic prescription (53.5%), lack of access to local antibiogram data (12.3%) and prescriber poor awareness about AMR (9.2%). Factors perceived for excessive antibiotic prescriptions were: patient drive (56%), treatment failure (79%), unknown febrile illnesses (39.7%) and upper respiratory tract infections (33.4%).

**Conclusion:**

Majority of physicians and nurses lack up to-date knowledge on AMR. Unavailability of local antibiogram data, self-prescription by patients and poor awareness on AMR are areas of interventions for prevention and control of AMR.

## Background

Antimicrobial resistance (AMR) is a growing serious worldwide public health problem in both hospital and community acquired infections [1]. Antimicrobial resistant bacteria have negative impact on treatment outcomes such as prolonged morbidity, hospital stay and increased risk of mortality [1,2]. Furthermore, patients infected with drug resistant bacteria demand more expensive therapy. Therefore, AMR results in increased health care costs and financial burden to families and societies [2,3]. Antimicrobial resistance problem is challenging in low-income countries because of high prevalence of infection, irrational uses of antimicrobials, over-the-counter availability of antibiotics and lack of clinical microbiology laboratories for antimicrobial susceptibility testing [4].

Development of AMR is accelerated by excessive antimicrobial prescription [5]. More than 50% of antibiotics worldwide are purchased without prescription [6]. The situation is more serious in developing countries because of antibiotic use without medical guidance and inadequate regulation of antibiotics [7]. Determinants for self-antibiotic prescription in low-income countries include over-the-counter sales of antibiotics, high cost of medical consultations and dissatisfaction with medical practitioners [8].

Containment of antimicrobial resistance requires change in the antimicrobial prescribing behavior of health workers. Changes in antimicrobial prescribing patterns will demand changes in physicians’ behavior towards the magnitude of AMR problem. Nurses in hospitals play important role in prevention of transmissions of resistant bacteria and promoting awareness on AMR for patients and communities. Thus, information on physicians’ and nurses’ knowledge and belief on AMR will permit the development of more effective interventions on containment of AMR. Surveys have been conducted to assess physicians’ knowledge and beliefs about antimicrobial use and resistance in USA and Europe [9-12]. However, these results are not necessarily applicable to the situation in low-income countries like Ethiopia.

The purpose of this study was to assess the knowledge and beliefs of physicians and nurses on AMR from 13 selected hospitals from public and private sectors in Amhara National Regional State (ANRS), Ethiopia. To our knowledge, this is the first study undertaken to assess the knowledge and beliefs about antimicrobial resistance among phycians and nurses in Ethiopia. The information generated in this study would be instrumental in planning and implementing preventive and control interventions on AMR at regional and national levels.

## Methods

### Study design, period and setting

A cross-sectional survey was conducted among eligible physicians and nurses working in hospitals in June 2013. Five in private and eight public hospitals were selected using systematic selection method by calculating the sampling interval. At the time of the survey, 235 physicians and 4,902 nurses were rendering services in a total of 26 hospitals (19= public and 7 private) in ANRS [13].

A sample size of 411 was calculated using the Epi info 7.0 soft ware (CDC, Atlanta, USA) considering a total population of 5,137 physicians and nurses. Expected maximum correct answer on the questions about knowledge of AMR (50%) was used. A 95% confidence level and 15% non-response were employed. Medical doctors from psychiatry, radiology, ophthalmology and anaesthesiology were excluded because they do not regularly prescribe antimicrobials. Participants, based on the number of physicians and nurses in each hospitals were selected by simple random sampling using lottery technique. However, in hospitals where the numbers of participants were less than five physicians, all participants were included.

### Data collection instruments

Prior to data collection the questions were piloted at local hospital. A total of 34-item-questions were self-administered to survey professional profiles, knowledge and beliefs on antimicrobial resistance.

The seven-item questions were used to address professional profiles such as qualifications, speciality, working hospital departments, service years, sources of information on antimicrobial resistance, training on AMR, exposure of using antimicrobial susceptibility test (AST) results and working in public and private hospitals.

Participants’ knowledge on AMR was surveyed by 13 item-questions. Lists of 7-item-questions addressing factors for development and spread of drug resistance. In addition, one open ended question was included to assess the perception of participants on factors promoting AMR at local and national levels. Three questions were used to assess their knowledge on scope of AMR problem from local to global levels. Two open ended questions were used to assess their knowledge about prevalence of drug resistant bacteria and hospital infections related to drug resistance.

Participants’ beliefs on AMR were assessed by 14-item questions. A list of seven questions and one open ended question covering the potential interventions to reduce antimicrobial resistances had been used. Lists of five questions were applied to assess beliefs towards rational use of antibiotics. One open ended question was used to ask participants to mention the least effective antibiotic in their hospital practices.

### Statistical analysis

The response alternatives for knowledge items were dichotomous. The questions on beliefs used Likert-style responses. Data was entered and analyzed using the Statistical Package for Social Sciences (SPSS® 20.0, USA). Chi-square test was used to assess the differences between physicians and nurses on knowledge and beliefs on AMR. P value of <0.05 (two sided) was taken as statistical significance.

### Mean score

For knowledge assessment, each correct response was given a score of 1 while a wrong or doubtful response was scored as 0. For both study participants mean knowledge scores < 0.72 were considered as below the expected level of knowledge while average scores ≥0.72 were at the expected level of knowledge. Regarding beliefs, useful and very useful responses were considered as positive beliefs. Scores from 0.15 to 0.88 were interpreted as negative beliefs, and scores ranged from 0.88 to 1 were considered as positive beliefs.

### Ethical consideration

Ethical clearance was obtained from the Research and Ethical Review Board of Bahir Dar University. Moreover, all patients gave written informed consent to participate in this study.

## Results

### Participants’ profiles and sources of information on AMR

A total of 385 participants took part in this study; of whom, 37 (9.6%) were senior physicians, 138 (35.8%) general practitioners and 210 (54.5%) nurses (Table 1). The overall response rate of participants was 91.4%. Of these, 175 (87%) physicians and 100% nurses filled the self-administered questionnaires. All study participants from private hospitals completed the questionnaires (response rate 100%). The overall mean service year was 7.2 (SD ± 8.2). The majority of the participants were from public hospitals (Table 1).

**Table 1:** Profiles of participants in 13 selected hospitals in Amhara Region, Ethiopia

**Variables**	**Senior physicians (n = 37)**	**GP (N = 138)**	**Nurses (n = 210)**	**Total (n = 385)**
	N (%)	N (%)	N (%)	N (%)
Work place				
Public only	13 (35.2)	124 (89.9)	171 (81.4)	308 (80)
Private only	12 (32.4)	0	36 (17.1)	48 (12.5)
Both in Public and Private	12 (32.4)	14 (10.1)	3 (1.5)	29 (7.5)
Hospital department				
Medicine	11 (29.7)	58 (42)	75 (35.7)	144 (37.4)
Surgery	15 (40.5)	37 (26.8)	54 (25.7)	106 (27.5)
Paediatrics	3 (8.1)	20 (14.5)	45 (21.4)	71 (18.4)
Gynae-Obstatrics	7 (18.9)	13 (9.4)	15 (7.1)	35 (9.0)
Rotation (among wards)	0	10 (7.2)	21 (10)	31 (8.0)
Training attended on AMR	15 (40.5)	12 (8.7)	9 (4.3)	36 (9.3)
Exposure of using AST results	20 (53.1)	64 (46.4)	48 (22.8)	132 (34.3)
Source of information				
Books	15 (40.5)	46 (33.3)	15 (7.1)	76 (19.7)
Internet	6 (16.2)	7 (5)	0	13 (3.4)
Journals	7 (18.9)	4 (2.9)	0	11 (2.8)
School course and workshops	4 (10.8)	40 (28.9%)	51 (24.4)	95 (24.6)
Lack of up- to -date information	5 (13.5)	41 (29.7)	144 (68.5)	190 (49.3)

Key: GP: General Practitioner Medical Doctors.

Senior physicians: Medical doctors with specializations.

Participants’ exposure of using antimicrobial susceptibility test (AST) result, training status on AMR and working departments are illustrated in (Table 1). Of the study participants, 13.5% of senior physicians, 29.7% general practitioners and 49.3% of nurses replied that they had no up-to-date information about AMR.

Participants were asked to mention their current sources of information about AMR. Overall, 19.7% respondents got information from relevant books, 3.4% from internet, 2.8% journals and 26.4% from other sources such as college or university courses and trainings. Regarding training need, 65% of physicians and 98% of nurses responded that they need further training on stewardship of antimicrobial resistance.

### Knowledge on antimicrobial resistance

The overall mean knowledge score was 0.72 (SD ± 0.44). The mean knowledge score was 0.82 (SD ± 0.37) for physicians and 0.63 (SD ± 0.40) for nurses (p = 0.001). Thus, 278 (72.2%) of participants were at the expected level of knowledge on AMR. Of these, 145 (82.8%) of physicians and 133 (63.5%) of nurses were knowledgeable (p = 0.001). Physicians’ and nurses’ knowledge about the scope of antimicrobial resistance problem at local, national and global levels are depicted in Table 2.

**Table 2:** Percentage of physicians and nurses rating the scope of AMR problem

**Scope of antimicrobial resistance**		**Strongly agree**	**Agree**	**Disagree**	**Strongly disagree**	**Don’t know**
AMR is worldwide problem	Physicians	39	43.0	9.8	4.6	2.9
	Nurses	24.8	35.2	26.4	4.6	10
	Total	31.5	39.0	18.1	4.6	6.3
AMR is problem in Ethiopia	Physicians	51.3	36.8	3.9	0	8.9
	Nurses	37.1	52.8	7.1	1.4	1.4
	Total	42.0	49.3	4.9	0.5	3.3
AMR is a problem in your hospital	Physicians	25	50.0	18.4	1.3	6.5
	Nurses	22.3	53.8	17.1	2.8	4.3
	Total	23.6	52.0	17.8	1.9	4.6

### Knowledge on causes and prevalence of antimicrobial resistance

According to the respondents the most important perceived factors contributing for antimicrobial resistance development were: Patients’ poor adherence to prescribed antibiotics (86% by respondents), widespread or overuse of antibiotics (80%) and broad spectrum antibiotics use (78.4%). Statistically significant difference was observed between physicians and nurses in rating factors for developing AMR (Table 3).

**Table 3:** Physicians’ and nurses’ knowledge about the causes of antibiotic resistances and antibiotic resistant bacteria

**Variables**	**Physicians (n = 175)**	**Nurses (n = 210)**	**Total**	**P-value**
	**Yes N (%)**	**Yes N (%)**	**Yes N (%)**	
**Causes of AMR**				
Widespread or over use of antibiotics promotes AMR	162 (92.5)	147 (70)	309 (80.5)	0.001
Usage of broad- spectrum antibiotics promote AMR	155 (88.5)	147 (70)	302 (78.4)	0. 001
Bacterial mutations cause of AMR	144 (82.3)	132 (62.8)	276 (71.6)	0.001
Poor hand washing practice in hospitals spread AMR	68 (38.8)	90 (42.8)	158 (41)	0.56
Poor infection control in hospitals spread AMR	104 (59.4)	114 (54.3)	218 (56.6)	0.40
Patient poor adherence promote AMR	160 (91.4)	171 (81.4)	331 (86)	0.002
Sub-standard quality of antibiotics	138 (78.8)	158 (75.2)	296 (76.8)	0.42
**Examples of antibiotic resistant bacteria in hospitals**				
Methicillin resistant *S. aureus* (MRSA)	39 (22.3)	5 (2.5)	44 (11.4)	0.001
MDR-TB	7 (4.1)	85 (40.4)	92 (23.9)	0.001
*P. aeruginosa*	10 (5.7)	0	10 (5.7)	
**Local factors for development of AMR**				
Self-prescription by patients	133 (76.0)	73 (34.7)	206 (53.5)	0.001
Lack of access to local antibiogram data	28 (16.0)	19 (9.0)	47 (12.3)	0.04
Prescribers’ poor awareness on AMR	27 (15.4)	8 (3.8)	35 (9.2)	0.001

Key: AMR: Antimicrobial resistances and MDR-TB: multi-resistant drug resistant tuberculosis.

Furthermore, assessment of knowledge on local factors for spread of AMR was also augmented by open ended question. The most important local factors identified were: self-antibiotic prescription was responded by 53.5%, lack of access to local antibiograms was responded by 12.3% and prescribers and patients poor awareness on AMR was responded by 9.2%.

Participants were also asked to rate bacterial infections in which drug resistant bacteria would be a problem in hospitals. Pneumonia was rated by 29.3% and 57.6% of physicians and nurses, respectively. Urinary tract infection was rated by 23.1% and 24.7% of physicians and nurses, respectively. Surgical site infection was rated by 19.5% and 20% of physicians and nurses, respectively. The interesting response was that 57.1% of nurses incorrectly perceived tuberculosis as the most prevalent hospital infections in which drug resistance is common but phycians rate tuberculosis by 2.3%.

Participants were asked to identify the most common drug resistant bacteria in hospitals they perceive from local to global levels by open question. Methicillin resistant *S.aureus* (MRSA) was mentioned by 22.3% and 2.4% of physicians and nurses, respectively. MDR-TB was listed by 40.4% of nurses and 4.1% physicians. *Pseudomonas aeruginosa* was rated by 5.7% of physicians (Table 3).

### Beliefs on potential interventions for antimicrobial resistance

Considering the mean score of beliefs (0.88), 95 (54%) of physicians and 109 (52%) of nurses believed in the suggested potential solutions for preventing AMR. Antimicrobial usage policy and reduction of antimicrobial usage for outpatients were not important by 32.4% and 38% of respondents, respectively (Table 4). Furthermore, the most frequently considered interventions were local access for antimicrobial susceptibility data (31.4%) and providing education on antimicrobial stewardship (17.2%).

**Table 4:** Physicians’ and nurses’ beliefs on potential intervention to combat AMR

**Variables**		**Very useful**	**Useful**	**Not useful**	**Not sure**
Antimicrobial usage policy	Physicians	26.4	40	18.2	15.4
	Nurses	30.0	36.8	20.5	10.5
	Total	28.2	38.4	19.4	13
Reduction of antibiotic use for outpatient setting	Physicians	15.3	59.4	18.8	5.9
	Nurses	33.3	47.0	12.6	7.3
	Total	24.2	53.2	31.4	6.6
Establish national AMR surveillance	Physicians	66.4	31.7	1.1	0.6
	Nurses	56.8	37.3	3.6	2.1
	Total	61.6	34.5	2.5	1.3
Establish hospital infection control committee	Physicians	54.7	42.3	2.3	0.6
	Nurses	55.3	42.2	0	2.4
	Total	55.0	42.2	2.3	3
Develop institutional guideline for antimicrobial use	Physicians	73.0	25.8	1.1	0
	Nurses	63.1	31.5	0	5.2
	Total	68.0	28.6	1.1	5.2
Education on antimicrobial therapy for prescribers	Physicians	68.8	28.8	1.1	1.1
	Nurses	53.6	40.5	3.6	2.1
	Total	61.2	34.6	2.3	1.6
Establish microbiology diagnostic services	Physicians	67.6	24.1	0	4.7
	Nurses	57.3	33.1	0	9.4
	Total	62.4	28.6	0	7.0

### Beliefs on misuse of antimicrobials

Physicians’ and nurses’ beliefs towards the pushing factors for frequent prescription of antibiotics were significantly different (Table 5). For both physicians and nurses, the most leading factors were: patients drive on prescribers to prescribe antibiotics, treatment failure and critically ill patients. Unknown febrile illness and upper respiratory infections were identified as important factors for excessive antibiotic prescriptions by 53.7% and 44.7% of respondents, respectively.

**Table 5:** Physicians’ and nurses’ belief on causes of unnecessary antibiotic prescriptions

**Questions**	**Physicians (n = 175)**	**Nurses (n = 210)**	**Total**	**P value**
	Yes (%)	Yes (%)	Yes (%)	
Patient push	61.0	53	56.7	0.04
Treatment failure	90.2	69.5	79.0	0.001
Critically ill or immune-compromised patient	52.3	67.1	60.4	0.01
Profit of hospitals	35.6	41	0.55	38.5
For which infections do you think unnecessary antibiotics would be prescribed?				
Upper respiratory tracts	44.7	24	33.4	0.001
Unknown febrile illness	53.7	28	39.7	0.001
Urinary tract infections	9.3	34.8	23.1	0.001
Diarrhoea	21.3	28	25.4	0.08

Participants’ beliefs towards the least effect antibiotics due to resistant bacteria were: amoxicillin rated by 36.5% of respondents, penicillin and cloxacillin by 22% each, chloramphenicol (16%) and cotrimoxazole (14%). Statistically significant difference was noted between physicians’ and nurses’ beliefs with regard to effectiveness of amoxicillin and cloxacillin (p = 0.001). Because, 28% of physicians and 8.5% of nurses mentioned amoxicillin as least effective antibiotics (p = 0.001). Cloxacillin was perceived as least effective by 14% of physicians and 8% of nurses (p = 0.01).

## Discussion

Physicians and nurses are key stakeholders in prevention and control of AMR through prescribing antimicrobials wisely, controlling transmission of drug resistant bacteria and promoting awareness. Thus, the present study demonstrated the knowledge and beliefs of physicians and nurses about AMR from 13 selected hospitals in ANRS, Ethiopia.

Statistically significant difference was observed between physicians and nurses in some aspects of knowledge and beliefs on AMR (p = 0.001) (Table 3 and Table 5). Regarding to awareness on the scope of AMR problem, majority of respondents strongly agreed or agreed AMR as global and national public health problems. However, few participates recognized AMR as a problem in their won hospital. These findings are consistent with a study conducted in Sudan [14], India [15] and DR Congo [16]. In contrast, previous studies from Spain, Brazil and Peru showed that more than 90% of physicians perceived AMR as a global and national problem [17-19].

The majority of physicians and nurses did not mention the existence of antibiotic resistant bacteria. For instance, only 22.3% of physicians and 2.4% of nurses had information regards to MRSA. These are lower than physicians’ knowledge of MRSA documented from India [15]. Although, MDR-TB is not a hospital pathogen, 40.4% of nurses mentioned it as one of the most prevalent drug resistant bacteria in hospitals. These knowledge gaps on local antibiotic resistant bacteria would be attributable to unavailability or lack of bacteriological culture and susceptibility testing in most hospitals [12]. Furthermore, only 9.3% of respondents have had training on antimicrobial stewardship education.

There was statistically significant difference between physicians and nurses with regard to the causes on development and spread of AMR (Table 3). The three leading causes of AMR were: patients’ poor adherence to prescribed antibiotics, over use of antibiotics and frequent prescription of broad-spectrum antibiotics. Likewise, a study conducted in Scotland, France and Spain stated that too many antibiotic prescriptions, too many broad-spectrum antibiotics and inappropriate duration of antibiotic treatments were the leading factors [19,20]. Poor hand washing was not well recognized in this study as contributing factors to AMR in hospital settings similar to other studies [19,20]. Therefore, emphasis on proper hand washing and infection control measures must be implemented.

This study revealed that the most important local factors for spread and development of AMR were self-antibiotic prescription and patient poor adherence to prescribed antimicrobial agents. Moreover, lack of access to antibiotic susceptibility testing and prescribers’ poor awareness on AMR were mentioned as local factors. Some other studies also supported this specified findings [6,7,14,16].

Regarding potential interventions to combat AMR, majority of participants believed in the most the favourite measures were: establishing national AMR surveillance program, avail clinical microbiology laboratory and local guidelines for rational use of antibiotics (Table 4). However, only 66.6% and 77.4% of respondents favoured antibiotic restriction policy and reduction of antibiotic use for outpatients, respectively. This finding is in agreement with a previous report on beliefs of physicians from USA [11]. Establishing local antimicrobial susceptibility tests and providing education on antimicrobials stewardship for health professional were identified as the most important interventions [14,19].

Physicians’ and nurses’ beliefs towards the pushing factors for frequent prescription of antibiotics were significantly different (p = 0.001). Treatment failure and critically ill patients were the most driving factors. Patients push to antibiotics was also mentioned by 56.7% of respondents [16]. More than half of physicians believed that unknown febrile illness was one of first factors for excessive antibiotic prescriptions.

According to the respondents, amoxicillin, penicillin and cloxacillin were perceived as the least effective antibiotics related to drug resistant bacteria. This perception could be considered as positive towards the recognition of drug resistance. Because, previous studies in the same region showed that bacterial isolates from clinical samples revealed high levels of resistance against these antimicrobials [21-23]. Furthermore, studies revealed that amoxicillin and cloxacilin are the most commonly prescribed antibiotics in different part of Ethiopia [24,25].

The major strength of this survey was large sample size and inclusion of 13 selected hospitals from public and private sectors. However, this study could not analyze the knowledge and belief differences among participants working in private and public hospitals because 32.4% of senior physicians and 10% general practitioners were working both in public and private-owned hospitals. Furthermore, participants’ prescribing practices and knowledge on the exact prevalence of antimicrobial resistant bacteria was not assessed because there was no national antimicrobial surveillance program. As general limitation of knowledge and belief survey, study participants may have tendency to provide socially desirable responses.

## Conclusion

This study revealed important information on knowledge and beliefs of physicians and nurses about AMR that would be implemented in a low-income country like, Ethiopia. In this survey, physicians and nurses working in hospitals had information gap on antimicrobial resistance. According to participants’ response, unavailability of local antibiogram data linked with self- drug prescription by patients and poor awareness on AMR are an issue.

## Competing interests

The authors declare that they have no competing interests.

## Authors’ contributions

AB designed the study, collected data, analyzed data and wrote the article. KM, reviewed the study plan, questionnaires and the article. MW analyzed data and reviewed the manuscript. All authors read and approved the final manuscript.

## Pre-publication history

The pre-publication history for this paper can be accessed here:

http://www.biomedcentral.com/2050-6511/15/26/prepub
